# Spontaneous Mesenteric Hematoma Secondary to a Pancreaticoduodenal Artery Aneurysm in Median Arcuate Ligament Syndrome: A Case Report

**DOI:** 10.7759/cureus.109431

**Published:** 2026-05-22

**Authors:** Moulayemin Beddy, Brahim El Mahjoub, Mokhtar El Mekhtoume, Hajar Ouazzani, Ismail Chaouche, Amal Akammar, Nizar El Bouardi, Meriem Haloua, Badreddine Alami, Meryem Boubbou, Mustapha Maaroufi, Y. Lamrani

**Affiliations:** 1 Department of Radiology, University Hospital Hassan II, Fez, MAR; 2 Department of Radiology, University Hospital Hassan II, Sidi Mohamed Ben Abdellah University, Fez, MAR; 3 Department of Mother and Child Radiology, University Hospital Hassan II, Sidi Mohamed Ben Abdellah University, Fez, MAR; 4 Department of Mother and Child Radiology and Interventional Imaging, University Hospital Hassan II, Fez, MAR

**Keywords:** celiac artery stenosis, coil embolization, endovascular management, median arcuate ligament syndrome (mals), mesenteric hematoma, pancreaticoduodenal artery aneurysm, spontaneous visceral aneurysm rupture

## Abstract

We report the case of a 45-year-old heavy smoker (20 pack-years) presenting with acute abdominal pain. Physical examination revealed diffuse abdominal tenderness without hemodynamic instability. Contrast-enhanced abdominal CT angiography demonstrated a large mesenteric hematoma measuring 24 × 25 × 11 cm, associated with a ruptured inferior pancreaticoduodenal artery aneurysm. It also showed significant celiac axis stenosis secondary to extrinsic compression by the median arcuate ligament, with post-stenotic dilatation, consistent with a hemodynamic alteration of the pancreaticoduodenal arcade. Emergency selective angiography confirmed active hemorrhage arising from the pancreaticoduodenal arcade. Urgent superselective coil embolization was performed, achieving effective hemostasis without periprocedural complications. The clinical course was favorable, with rapid symptom resolution and uneventful recovery. This case highlights the critical role of CT angiography in identifying both the bleeding source and the underlying vascular abnormality, and demonstrates the effectiveness of superselective transarterial embolization as a first-line treatment in this setting of celiac axis compression-related hemodynamic disturbance.

## Introduction

Median arcuate ligament syndrome (MALS), also known as celiac trunk compression syndrome, is a rare entity caused by extrinsic compression at the origin of the celiac trunk by the median arcuate ligament of the diaphragm [1]. This compression reduces blood flow in the celiac trunk territory, leading to compensatory circulation through the pancreaticoduodenal arcades from the superior mesenteric artery (SMA). The chronic increase in flow through these fragile arcades promotes the development of true high-flow aneurysms that are at high risk of spontaneous rupture [2]. We report the case of a patient presenting with sudden epigastric pain revealing a mesenteric hematoma secondary to the rupture of an aneurysm of an inferior pancreaticoduodenal branch in the setting of MALS.

## Case presentation

A 45-year-old male, a chronic heavy smoker (20 pack-years), with no significant past medical or surgical history and no regular medication use, presented to the emergency department with acute abdominal pain evolving over several hours. The pain was diffuse, progressively increasing in intensity, and was not associated with nausea, vomiting, or signs of gastrointestinal bleeding.

On admission, the patient was hemodynamically stable. Physical examination revealed diffuse abdominal tenderness without guarding or rebound. No palpable abdominal mass was detected. Initial laboratory workup, including hemoglobin level (11 g/dL) and inflammatory markers, was within normal limits.

Given the persistence of symptoms, a contrast-enhanced abdominal CT scan was performed. Imaging demonstrated a large mesenteric hematoma measuring approximately 24 × 25 × 11 cm, appearing spontaneously hyperdense with a mean attenuation of 60 Hounsfield units, consistent with acute to subacute hemorrhage.

**Figure 1:**
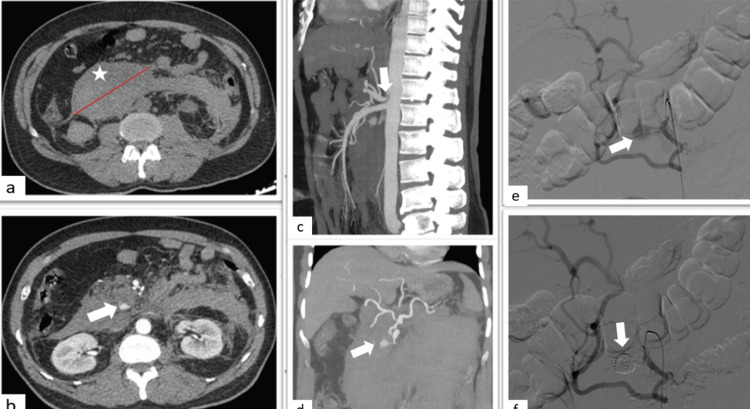
Abdominal CT and diagnostic/therapeutic mesenteric angiography in a case of spontaneous mesenteric hematoma secondary to a pancreaticoduodenal artery aneurysm with median arcuate ligament compression. Multiphase abdominal CT angiography shows a large spontaneously hyperdense mesenteric hematoma measuring approximately 24 cm (a) and an aneurysm arising from a branch of the pancreaticoduodenal arcade originating from the superior mesenteric artery (arrows in b and d). Sagittal arterial-phase reconstruction demonstrates moderate compression of the celiac trunk by the median arcuate ligament (c). Selective mesenteric angiography confirms the aneurysm (arrow in e), which was successfully treated by coil embolization (arrow in f).

CT angiography of the abdomen revealed a saccular aneurysm arising from a branch of the inferior pancreaticoduodenal artery originating from the SMA. The aneurysm measured 16 × 12 mm with a wide neck measuring 8 mm (neck-to-sac ratio of 0.5) and no major branches arising from the sac. In addition, a focal stenosis at the origin of the celiac trunk was observed, secondary to extrinsic compression by the median arcuate ligament, associated with post-stenotic fusiform dilatation (Figure 1, Panels a-d).

The overall imaging findings were consistent with MALS complicated by a ruptured inferior pancreaticoduodenal artery aneurysm resulting in a large mesenteric hematoma, a well-documented complication in the literature.

The patient subsequently underwent endovascular treatment. After right femoral artery puncture and placement of a 5-F introducer sheath, selective catheterization of the SMA was performed using a 5-F Cobra C2 catheter. Angiography revealed multiple anastomotic arterial branches arising from the pancreaticoduodenal arcade supplying the celiac trunk territory, along with a saccular aneurysm arising from an inferior pancreaticoduodenal branch. Selective microcatheterization of this branch was achieved using an Echelon microcatheter advanced over a Runthrough microguidewire (Figure 1, Panel e). Embolization was then performed by deploying four coils within the aneurysmal sac (three 12 mm × 40 cm coils and one 14 mm × 51 cm coil), followed by occlusion of the proximal parent artery using two additional coils (one 5 mm × 8 cm coil and one 9 mm × 20 cm coil). Final control angiography confirmed complete exclusion of the aneurysm with no residual filling (Figure 1, Panel f).

The clinical course was favorable, with complete resolution of abdominal pain and no evidence of post-procedural complications.

## Discussion

MALS classically manifests with postprandial epigastric pain, nausea, vomiting, and weight loss. It predominantly affects young patients (mean age of approximately 30 years) with a female predominance. However, most cases of anatomical celiac artery compression remain asymptomatic, with only 10-25% of patients developing significant clinical symptoms [3].

Celiac artery stenosis induces compensatory retrograde flow through the pancreaticoduodenal arcades, subjecting these vessels to significant hemodynamic stress. This explains the formation of true aneurysms, particularly of the pancreaticoduodenal arteries, which represent the main collateral network between the celiac and superior mesenteric territories. Although rare (accounting for approximately 2% of all visceral artery aneurysms), these aneurysms are associated with celiac axis stenosis or occlusion in nearly half of the cases. The median arcuate ligament is involved in 10-30% of these situations [4].

Radiologically, the diagnosis of MALS is based on the demonstration of celiac trunk stenosis with a characteristic “hooked” appearance on sagittal CT or MR reconstructions. Post-stenotic dilatation and the development of collaterals from the SMA are frequently observed. Dynamic imaging during end-inspiration and end-expiration helps differentiate fixed compression (persistent or worsened on expiration) from transient physiological compression. Catheter angiography remains the gold standard for dynamic assessment of the degree of stenosis and retrograde filling of the celiac axis. Doppler ultrasound can help quantify the stenosis, with a peak systolic velocity greater than 200 cm/s corresponding to ≥70% stenosis [5].

Pancreaticoduodenal artery aneurysms carry a high risk of rupture (up to 60-65% of reported cases), independent of their size. Rupture may present as a retroperitoneal or mesenteric hematoma, sometimes massive, as observed in our patient. In the literature, more than half of reported cases are diagnosed at the time of rupture, with significant mortality when management is delayed [6].

The management of ruptured pancreaticoduodenal artery aneurysms has shifted toward an endovascular-first approach (coil embolization or other occlusive agents), with open surgery reserved for cases of technical failure or complex anatomy [7]. Several studies suggest that celiac artery revascularization or median arcuate ligament release may not always be necessary after successful aneurysm exclusion, particularly in patients who become asymptomatic following the acute episode [8]. Other authors advocate a staged approach with urgent embolization followed by ligament release [9]. In some cases of rupture, surgical resection of the median arcuate ligament has been performed after embolization to prevent recurrence [10].

## Conclusions

We report a case of a pancreaticoduodenal artery aneurysm associated with MALS, a rare but life-threatening cause of acute abdominal pain. Contrast-enhanced CT angiography enabled rapid diagnosis by identifying a large mesenteric hematoma, a ruptured inferior pancreaticoduodenal artery aneurysm, and associated celiac axis compression. Emergency transarterial coil embolization achieved immediate and complete exclusion of the aneurysm, with prompt hemodynamic stabilization and full clinical recovery. This case highlights the decisive role of CT angiography for early diagnosis and the effectiveness of endovascular embolization as a life-saving first-line treatment.
